# Endovascular renal sympathetic denervation to improve heart failure with reduced ejection fraction: the IMPROVE-HF-I study

**DOI:** 10.1007/s12471-021-01633-z

**Published:** 2021-10-05

**Authors:** L. Feyz, R. Nannan Panday, M. Henneman, F. Verzijlbergen, A. A. Constantinescu, B. M. van Dalen, J. J. Brugts, K. Caliskan, M. L. Geleijnse, I. Kardys, N. M. Van Mieghem, O. Manintveld, J. Daemen

**Affiliations:** 1 University Medical Center, Department of Cardiology, Erasmus MC, Rotterdam, The Netherlands; 2 Department of Radiology and Nuclear Medicine, Erasmus MC, University Medical Center, Rotterdam, The Netherlands; 3 Department of Radiology and Nuclear Medicine, Radboud University Medical Center, Nijmegen, The Netherlands; 4 Department of Cardiology, Franciscus Gasthuis & Vlietland, Rotterdam, The Netherlands

**Keywords:** Heart failure, Iodine-123 meta-iodobenzylguanidine, Sympathetic overactivity, Renal sympathetic denervation

## Abstract

**Introduction:**

The aim of the present study was to assess the safety and efficacy of renal sympathetic denervation (RDN) in patients with heart failure with reduced ejection fraction (HFrEF).

**Methods:**

We randomly assigned 50 patients with a left ventricular ejection fraction (LVEF) ≤ 35% and NYHA class ≥ II, in a 1:1 ratio, to either RDN and optimal medical therapy (OMT) or OMT alone. The primary safety endpoint was the occurrence of a combined endpoint of cardiovascular death, rehospitalisation for heart failure, and acute kidney injury at 6 months. The primary efficacy endpoint was the change in iodine-123 meta-iodobenzylguanidine (^123^I‑MIBG) heart-to-mediastinum ratio (HMR) at 6 months.

**Results:**

Mean age was 60 ± 9 years, 86% was male and mean LVEF was 33 ± 8%. At 6 months, the primary safety endpoint occurred in 8.3% vs 8.0% in the RDN and OMT groups, respectively (*p* = 0.97). At 6 months, the mean change in late HMR was −0.02 (95% CI: −0.08 to 0.12) in the RDN group, versus −0.02 (95% CI: −0.09 to 0.12) in the OMT group (*p* = 0.95) whereas the mean change in washout rate was 2.34 (95% CI: −6.35 to 1.67) in the RDN group versus −2.59 (95% CI: −1.61 to 6.79) in the OMT group (*p*-value 0.09).

**Conclusion:**

RDN with the Vessix system in patients with HFrEF was safe, but did not result in significant changes in cardiac sympathetic nerve activity at 6 months as measured using ^123^I‑MIBG.

**Supplementary Information:**

The online version of this article (10.1007/s12471-021-01633-z) contains supplementary material, which is available to authorized users.

## What’s new?

- In patients with HFrEF, renal sympathetic denervation (RDN) did not result in a significant change in cardiac sympathetic nerve activity using specific ^123^I‑MIBG nuclear imaging
- RDN appeared safe with the Vessix system, with no effect on blood pressure in patients with HFrEF
- NYHA class worsened significantly in the optimal medical therapy group at follow-up indicating the progressive nature of congestive heart failure
- A third of the patients in the RDN group improved to NYHA I
- Conducting larger and sham-controlled studies, assessing the effect of RDN on left ventricular performance and quality of life is warranted

## Introduction

Chronic heart failure is a major public health problem, with a prevalence of 1–2% in the adult population [1]. While pharmacological treatment for heart failure with reduced ejection fraction (HFrEF) has shown to prevent hospitalisation and improve quality of life and survival, its long-term prognosis remains poor justifying a persistent need for novel therapeutic strategies that improve both morbidity and mortality [2–6].

Increased sympathetic tone has been directly linked to severity of heart failure and adverse outcome [7, 8]. In response to a chronic low-output state in HFrEF, neurohormonal adaptations occur such as the activation of the renin-angiotensin-aldosterone-system (RAAS) and the sympathetic nervous system (SNS) in order to maintain vital organ perfusion [9, 10].

In the past decade, renal sympathetic denervation (RDN) emerged as a novel minimally invasive treatment option to reduce sympathetic tone and proved to significantly reduce blood pressure in hypertensive patients [11–14]. Promising findings were subsequently reported on the effects of RDN in HFrEF animal models [15, 16]. Up to now, the clinical evidence for RDN in the treatment of HFrEF is limited and restricted to several small non-randomised studies [17, 18]. In contrast to several studies with pharmaceutical agents, data correlating the effect of RDN on cardiac sympathetic tone as measured using iodine-123 labelled meta-iodobenzylguanidine (^123^I‑MIBG) is lacking. The present study aimed to assess the safety and efficacy of RDN in patients with HFrEF as measured using ^123^I‑MIBG at 6 months.

## Methods

This present study is a single centre open label prospective randomised controlled trial designed to allocate 70 patients to treatment with RDN and optimal medical therapy (OMT) or OMT alone (1:1).

Due to the impact of several studies disputing the effect of RDN in patients with arterial hypertension, subsequent slow inclusion and the decision of the manufacturer of the device to halt further production of the Vessix V2 Renal Denervation System (Boston Scientific, Natick, MA, USA), inclusion was halted after the first 50 patients. This study was approved by our local ethics committee and all patients provided written informed consent (*trialregister.nl, NTR number: NTR5328).*

Patients were eligible for enrolment when the following inclusion criteria were met: left ventricular ejection fraction (LVEF) ≤ 35% (as assessed by echocardiography), New York Heart Association (NYHA) functional class ≥ II, age between 18 and 75 years, renal arteries suitable for RDN (i.e. baseline diameter stenosis < 30%, main renal artery diameter of ≥ 3.5 mm and ≤ 7.0 mm for each kidney), a glomerular filtration rate (eGFR) of > 30 ml/min/1.73 m^2^. Exclusion criteria included: systolic office blood pressure < 110 mm Hg, recent (< 3 months) stroke or myocardial infarction, acute heart failure (HF), presence of other medical diseases or conditions associated with a life expectancy of less than one year.

Work-up at baseline included laboratory analyses, 24 h ambulatory blood pressure measurement (24 h ABPM), echocardiography, ^123^I‑MIBG, as well as a computed tomography (CT) scan to confirm renal artery eligibility. Clinical follow-up occurred at 1, 3 and 6 months and will be continued yearly up to 5 years. Follow-up renal artery imaging using CT was performed at 6 months in patients who underwent RDN.

### Study endpoints

The primary safety endpoint included the occurrence of a combined endpoint of cardiovascular death, rehospitalisation for heart failure, and acute kidney injury at 6 months. The primary efficacy endpoint was the change in ^123^I‑MIBG late heart-to-mediastinum ratio (HMR) at 6 months. Other safety parameters that were assessed at 6 months follow-up: major access site bleeding, change in renal function (measured in plasma: cystatin C and estimated by eGFR) and newly acquired renal artery stenosis and/or repeat renal artery intervention.

Secondary efficacy endpoints include (baseline vs 6‑month follow-up): change in NYHA class, 6‑minute walk test (6MWT), change in quality of life, echocardiographic endpoints, laboratory endpoints and change in diuretic dosage (based on a change in the defined daily dose, DDD) [19]. Quality of life and an overall physical and mental function survey (RAND-36 and the Kansas City Cardiomyopathy questionnaire (KCCQ)) were used at baseline and at 6‑month follow-up [20, 21]. All echocardiograms were assessed by dedicated imaging cardiologists unaware of the treatment allocation.

### ^123^I-MIBG scintigraphy data acquisition and analysis

For detailed data acquisition and analysis, our previous work should be used as a reference [22]. Calculation of WR was performed using the following formula (no correction for background): WR = (HMR_early_ − HMR_late_)/(HMR_early_) × 100% [23].

### RDN procedure

After administration of local anaesthesia, common femoral artery access was achieved by an ultrasound-guided puncture and a 6-Fr sheath was then introduced. Under fluoroscopic guidance, the short 6‑Fr sheath was exchanged for an 8‑Fr RDN or an IMA tipped guiding sheath, to accommodate the Vessix V2 Renal Denervation System. The Vessix V2 system consists of an over-the-wire balloon catheter and a radiofrequency generator. After engaging the renal arteries, selective renal artery angiograms were obtained and an appropriate balloon size was chosen (4 [4 electrodes] to 7 [6 electrodes] mm). Once balloon inflation was completed and the device activated, the generator raised the electrode temperature to 68 ºC—the temperature at which treatment is conducted—and nerve denervation was carried out within 30 s.

## Statistical analysis

The study was designed to compare the primary efficacy endpoint, late HMR and washout rate (WR) derived from ^123^I‑MIBG, in the treatment group versus control group (supplementary material for sample size calculation). Baseline categorical variables were expressed as counts and percentages. Differences in baseline categorical variables between randomly allocated treatment groups were compared using the chi-squared test, Fisher’s exact test or chi squared test for trend (NYHA class) when appropriate. Baseline continuous variables were described as mean ± standard deviation (SD) when normally distributed. In case of non-normal data distributions, data were presented as median [interquartile range, IQR]. Continuous variables (such as HMR and WR, normally distributed) were compared between groups using independent samples *t*-test or paired samples *t*-test. To examine within-group changes, paired samples *t*-tests were used. Non-parametric tests (Wilcoxon signed-rank or Mann Whitney U test, when appropriate) were used to analyse differences in case of non-normal distributions. All statistical tests are two-tailed. A *p*-value < 0.05 was considered statistically significant. Statistical analysis was performed using SPSS statistical analysis (version 24.0).

## Results

### Clinical characteristics

**Fig. 1:**
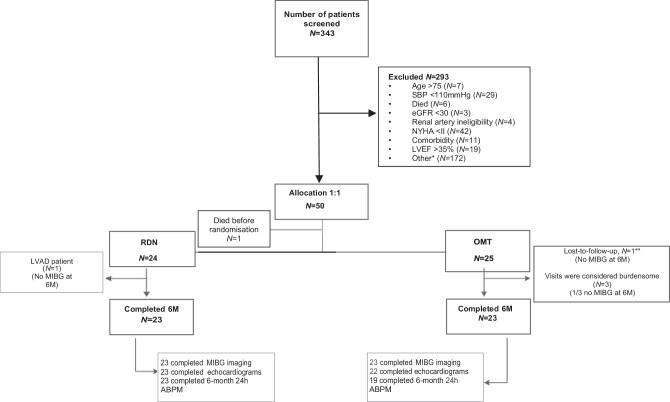
Patients screened for eligibility, *Other = participation in other research studies (*N* = 9), waiting for heart transplantation (*N* = 14), refused consent, due to study burden, (*N* = 31) or other reasons (*N* = 60), non-compliance (*N* = 5), distance to the hospital (*N* = 6), not yet on OMT (*N* = 23), unable to contact (*N* = 24). **Lost-to-follow-up (in the OMT-group) *N* = 1: patient retracted informed consent, still alive at 6 months. *ABPM* ambulatory blood pressure measurement,* eGFR* estimated glomerular filtration rate, *LVAD* left ventricular assist device, *LVEF* left ventricular ejection fraction, *MIBG* meta-iodobenzylguanidine,* NYHA* New York Heart Association, *OMT* optimal medical therapy, *RDN* renal sympathetic denervation, *SBP* systolic blood pressure, *6M* 6 months

A total of 343 patients were assessed for eligibility, 50/343 (14.6%) were enrolled between August 2014 and June 2018 (Fig. 1). There were no significant differences in patient characteristics, haemodynamic parameters and baseline medications between both groups at the time of inclusion (Tab. 1). Mean age was 60 ± 9 years, 86% was male, 78% in NYHA class II at baseline, ischaemic cardiomyopathy was present in 60% of the patients. Furthermore, mean baseline LVEF was 33 ± 8%, while mean left ventricular end-diastolic diameter (LVEDD) was 70 ± 11 mm. An implantable cardioverter-defibrillator (ICD)/cardiac resynchronisation therapy (CRT) device was present in 66% and 22% of patients respectively. Mean ambulatory blood pressure at baseline was 111/69 ± 13/7 mm Hg.

**Table 1:** Baseline characteristics

	RDN *N* = 24	OMT *N* = 25
Age, years	60 ± 8	59 ± 10
Male *n*, (%)	20 (83.3)	22 (88.0)
BMI, kg/m^2^	28.0 ± 4.4	27.9 ± 5.2
eGFR, ml/min	68.3 ± 17.6	69.8 ± 20.8
*ICD/CRT, (%)*	68/24	64/20
*Cardiomyopathy*		
iCMP *n*, (%)	15 (62.5)	14 (56.0)
DCM *n*, (%)	8 (33.3)	11 (44.0)
Other *n*, (%)	1 (4.2)	–
*Cardiovascular history (%)*		
Prior MI	12 (50.0)	13 (48.0)
Prior PCI	9 (37.5)	12 (48.0)
Prior CABG	5 (20.8)	–
CVA	3 (12.5)	1 (4.0)
*Cardiovascular risk factors (%)*		
Diabetes	6 (25.0)	10 (40.0)
Hypertension	14 (58.3)	10 (40.0)
Dyslipidaemia	18 (75.0)	15 (60.0)
Smoker, current	4 (16.7)	6 (24.0)
Family history of premature CVD	7 (29.2)	9 (36.0)
*Clinical parameters*		
24 h ABPM, mm Hg	111 ± 9/69 ± 6	108 ± 9/66 ± 5
Office BP, mm Hg	121 ± 11/75 ± 8	124 ± 19/75 ± 14
Heart rate, bpm	70 ± 9	67 ± 9
NYHA II, (%)	17 (70.8)	21 (84.0)
NYHA III, (%)	7 (29.2)	4 (16.0)
*Echocardiographic parameters*		
LVEF, %	32 ± 7	33 ± 9
LVEDD, mm	72 ± 7	69 ± 13
LVESD, mm	63 ± 8	61 ± 15
*Mean number diuretics, n (%)*	2 ± 1	2 ± 1
*Pharmacological therapy, n (%)*		
ACE-i/ATII-antagonist	15 (62.5)/7 (29.2)	21 (84.0)/4 (16.0)
Calcium channel blockers	2 (8.3)	2 (8.0)
Selective beta-blockers	21 (87.5)	23 (92.0)
Diuretics/MRA	20 (83.3)/21 (87.5)	25 (100)/19 (76.0)
Aspirin	12 (50.0)	14 (56.0)
Statins	17 (70.8)	18 (72.0)
*Procedural characteristics*		
Number ablations L/R, median [IQR]	11 [6–12]/10 [7–12]	–
Mean number of accessories L/R	2/1	–

Data was presented in mean ± SD or median [interquartile range, IQR] when appropriate

*ABPM* ambulatory blood pressure measurement, *ACE‑i* angiotensin-converting-enzyme inhibitor, *ATII* angiotensin-II antagonist, *BMI* body mass index, *BP* blood pressure, *CABG* coronary artery bypass graft, *CVA* cerebrovascular accident, *CVD* cardiovascular disease, *DCM* dilated cardiomyopathy, *eGFR* estimated glomerular filtration, *ICD/CRT* implantable cardioverter-defibrillator/cardiac resynchronisation therapy,* iCMP* ischaemic cardiomyopathy, *IQR* interquartile range, *LVEF* left ventricular ejection fraction, *LVEDD* left ventricular end-diastolic diameter,* LVESD* left ventricular end-systolic diameter*, MI* myocardial infarction, *MRA* mineralocorticoid receptor antagonist, *NYHA* New York Heart Association, *PCI* percutaneous coronary intervention

### Change in ^123^I-MIBG

No significant change was seen in late HMR and WR at 6 months between the RDN group and the OMT group respectively (Tab. 2). At 6 months, the mean change in late HMR was −0.02 (95% CI: −0.08 to 0.12) in the RDN group, versus −0.02 (95% CI: −0.09 to 0.12) in the OMT group (*p*-value for mean between group difference = 0.95), whereas the mean change in WR was 2.34 (95% CI: −6.35 to 1.67) in the RDN group versus −2.59 (95% CI: −1.61 to 6.79) in the OMT group (*p*-value for mean between group difference = 0.09).

**Table 2:** Change in ^123^I‑MIBG (primary efficacy endpoint)

	RDN		Difference (95% CI)	OMT		Difference (95% CI)	Mean between-group difference (95% CI)	*p-*value
	Baseline	6 months		Baseline	6 months			
Early HMR	2.14 ± 0.41	2.13 ± 0.43	−0.02 (−0.09 to 0.13)	2.44 ± 0.49	2.42 ± 0.48	−0.02 (−0.13 to 0.16)	0 (−0.18 to 0.18)	1.00
Late HMR	1.92 ± 0.43	1.90 ± 0.47	−0.02 (−0.08 to 0.12)	2.15 ± 0.47	2.13 ± 0.48	−0.02 (−0.09 to 0.12)	−0.004 (−0.14 to 0.13)	0.95
WR	11.3 ± 7.8	13.7 ± 8.2	2.34 (−6.35 to 1.67)	14.8 ± 11.5	12.2 ± 9.0	−2.59 (−1.61 to 6.79)	4.93 (−0.73 to 10.6)	0.09

^123^I‑MIBG is a physiologic analogue of norepinephrine and acts selectively on sympathetic nerve endings. By using cardiac neurotransmission imaging global information about neuronal function can be expressed in early, but more specifically in late HMR (reflecting the storage, regional distribution and release of ^123^I‑MIBG), with WR reflecting the neuronal integrity or sympathetic tone. Data was presented in mean ± SD, with differences presented in 95% CI

*HMR* heart-to-mediastinum ratio, *OMT* optimal medical therapy, *RDN* renal sympathetic denervation, *SD* standard deviation, *WR* washout rate

### Safety

The primary safety endpoint occurred in 2/24 patients in the RDN group (8.3%) vs 2/25 patients in the OMT group (8.0%) respectively (*p* = 0.97). In 3/24 (12.5%) patients, a minor access site bleeding was observed (all small haematomas with no further clinical consequences), no further peri-procedural complications occurred. In the RDN group, one patient received a left ventricular assist device (LVAD) due to refractory heart failure. Safety events are described in Tab. 3. eGFR remained unchanged in both cohorts; in the RDN group: 68 ± 17 ml/min at baseline vs 68 ± 20 ml/min at 6 months, *p* = 0.98. Similar findings were seen in the OMT group: 70 ± 19 ml/min vs 71 ± 21 ml/min, *p* = 0.94 (see Table S1 in Electronic Supplementary Material [ESM]).

**Table 3:** Safety endpoint

Endpoint	RDN	OMT
*Total events (%)*	*7/24 (29.2)*	*10/25 (40.0)*
^a^ Primary safety endpoint	2/24 (8.3)	2/25 (8.0)
*Specific events within 6M*		
– Death	0/24	0/25
– Myocardial infarction	0/24	2/25 (8.0)
– New-onset of ESRD	0/24	0/25
– Renal artery intervention	0/24	0/25
– Stroke	0/24	0/25
– Hospitalisation for HF	2/24 (8.3) ^b^	2/25 (8.0)
– Hospitalisation for AF	1/24 (4.2)	1/25 (4.0)
– Hospitalisation non-cardiac	0/24	2/25 (8.0) (colon carcinoma, non-Hodgkin lymphoma)
– New renal artery stenosis	1/24 (4.2)	0/25
– Side effects medication	2/24 (8.3) (statin-induced myalgia, amiodarone-induced hyperthyroidism)	–

*AF* atrial fibrillation, *ESRD* end-stage renal disease, *HF* heart failure, *VT* ventricular tachycardia

^a^ The primary safety endpoint includes the occurrence of a combined endpoint of cardiovascular death, rehospitalisation for heart failure and acute kidney injury at 6 months.

^b^ *N* = 1 received a LVAD (left ventricular assist device)

See supplementary online material for details on secondary endpoints.

## Discussion

RDN in patients with HFrEF did not result in a significant change in cardiac sympathetic nerve activity as measured using ^123^I‑MIBG late HMR and WR at 6 months. The therapy appeared safe. A significant difference was observed in LVEDD in the RDN group, and 26% of patients in the treatment group were in NYHA class I versus none in the control-group.

Percutaneous RDN was introduced about 10 years ago as a minimal invasive treatment option for patients with resistant hypertension, a condition linked to sympathetic overactivity [24]. Sympathetic overactivity proved to contribute to the progression of myocardial cell injury and left ventricular dysfunction in patients with HF and a significant correlation was found between the severity of overactivity and NYHA class [25]. As a result to the chronic low-output state in HFrEF, elevated sympathetic tone stimulates renin release by the kidneys, leading to sodium retention, volume expansion and renal vasoconstriction in order to maintain vital organ perfusion. However, due to a subsequent increase in peripheral resistance, myocardial contractility and increase in heart rate prognosis worsens. An inverse association was found between norepinephrine release and survival [26]. Human data from the REACH pilot study showed that RDN in seven patients with congestive HF was safe and associated with a significant increase in 6MWT [18]. A randomised study presented by Taborsky et al., showed that RDN in patients with more advanced heart failure (mean LVEF was 25%, *N* = 51) resulted in significant improvements in LVEF, left ventricular end-systolic diameter (LVESD) and LVEDD as well as in NT-pro-BNP while no change was seen in patients with OMT alone [17]. For reasons unknown, the study was never published.

To the best of our knowledge, our study is the first to assess the effect of RDN in patients with HFrEF using ^123^I‑MIBG imaging to assess cardiac sympathetic nerve activity. HMRs remained unchanged at 6 months in both arms. While we aimed to enrol patients with symptomatic HFrEF, the vast majority of the patients in our study were in NYHA class II with relatively low NT-pro-BNP values, suggesting a less severe HF phenotype. The latter could have explained part of the lack of effect in the present study and should be put into perspective due to the fact that our study was one of the first dedicated studies on the safety and efficacy of RDN in heart failure. Whether a more pronounced effect can be observed in patients with more advanced or unstable heart failure should be assessed in future dedicated studies.

The relatively low risk profile of our patients could also explain the higher than expected baseline HMRs in our study as compared to previous studies on the topic with baseline late HMRs in the range of 1.2 to 1.6 in patients with more pronounced heart failure and late HMRs of 2.5 ± 0.3 in healthy control [27]. The same applies for the WR found in our study which, being around 12%, were significantly below the threshold of 27% associated with poor prognosis [28]. This might suggest that the stable HF population studied in the present study was on relatively well controlled heart failure therapy in which the additional treatment with RDN did not add substantially on top of pre-existent OMT to improve cardiac sympathetic nerve activity.

Although our study was not powered to detect a difference in clinical endpoints, the overall rate of HF-related events at 6 months in the present study was low which might illustrate the relatively low risk profile of the patients included.

Based on the data available at the time of our study design, a significant blood pressure lowering effect of RDN was anticipated in patients with hypertension. This raised concerns about a potential blood pressure lowering effect of RDN in HF patients which might have forced down-titration of HF drugs. The latter made that we refrained from including patients with a baseline systolic blood pressure < 110 mm Hg and might have resulted in the HFrEF population at relatively low risk. Finally, in contrast to previous studies suggesting a significant change in LVEF following RDN, no change in LVEF was found in the present study. Conversely, we did observe a small, albeit significant, decrease in LVEDD following RDN. The latter results, however, should be interpreted with caution given the known variability in measurements derived from transthoracic echocardiography. However, we did observe a significant decrease in the peak late diastolic filling velocity in the treatment arm, which could implicate an improvement in left ventricular relaxation [29].

## Study limitations

The current study has a number of limitations. First, we enrolled a smaller number of patients than first intended due to slow inclusion rates. Therefore, the study was underpowered to reach its primary efficacy endpoint. Second, we cannot exclude the fact that we might have used a less efficacious RDN system. Whether the use of a different technology in the present study would have altered our findings remains unknown. Third, we included a lower risk HF phenotype (80% with NYHA II). Finally, the present trial was an open label trial and not sham-controlled.

## Conclusion

RDN with the Vessix system in patients with HFrEF was safe, but did not result in significant changes in cardiac sympathetic nerve activity at 6 months as measured using ^123^I‑MIBG.

## Supplementary Information

**Table S1. **Change in laboratory findings;** Table S2. **Change in echocardiographic parameters;** Table S3. **Change in blood pressure, heart rate and mean arterial pressure;** Table S4. **Defined Daily Dose (DDD) at 6 months within the groups; **Table S5. **Defined Daily Dose (DDD) at 6 months between the groups; **Table S6.** RAND-36 questionnaire
