# Supplementary material for: Endovascular renal sympathetic denervation to improve heart failure with reduced ejection fraction: the IMPROVE-HF-I study
Source: Neth Heart J. 2021 Oct 5;30(3):149–59. doi: 10.1007/s12471-021-01633-z (PMC8881518; doi:10.1007/s12471-021-01633-z)
Supplement: Supplementary file 1 — Table S1. Change in laboratory findings; Table S2. Change in echocardiographic parameters; Table S3. Change in blood pressure, heart rate and mean arterial pressure; Table S4. Defined Daily Dose (DDD) at 6 months within the groups; Table S5. Defined Daily Dose (DDD) at 6 months between the groups; Table S6. RAND-36 questionnaire [file 12471_2021_1633_MOESM1_ESM.docx]

**Supplementary material**

**Statistical analysis, sample size calculation**

The sample size was originally calculated at 33 patients per arm. To account for drop-outs, the proposed sample size for the present study was in total 70 patients. Assumptions were based on the OLOMOUC-I study, in which an increase in LVEF from 25 to 31% (SD 14%) was found at 1-year follow-up and the work of Kasama et al. showing an increase in H/M ratio from 1.64 ± 0.20 to 1.86 ± 0.27 and LVEF of 6% in patients with HFrEF following treatment with spironolactone[[17](#_ENREF_17), [24](#_ENREF_24)]. Eventually, 25 patients per arm were included; based on the assumptions above, this results in a power of 69%.

Results, secondary endpoints
NYHA class
As compared to baseline, NYHA class at 6 months remained unchanged in the RDN group, p=0.18, however in the OMT group functional class worsened significantly (p=0.002). At 6 months, 26.1% of the patients in the RDN group was in NYHA class I vs. none in the OMT group, p=0.03.

6MWT
The 6MWT remained unchanged at 6 months in both groups (RDN/OMT): +2.1m (95% CI: -29 to 24) vs. +0.1m (95% CI: -28 to 28), p-value for mean between group difference 0.91.

Laboratory parameters
No change was observed in NT-pro-BNP (pg/mL) at baseline vs. 6 months in the RDN group (19 [8 - 35] vs. 20 [9 - 47], p=0.83) vs. the OMT group (17 [10 - 24] vs. 15 [11 - 29], p=0.61), p-value for difference between group difference= 0.81 (Table S1).

Echocardiographic parameters
The mean change in LVEF at 6 months was +0.7% (95% CI: -2.7 to 1.3) in the RDN group as compared with +0.6% (95% CI: -2.5 to 1.3) in the OMT group (p-value for mean between group difference=0.96), whereas LVEDD significantly decreased in the RDN group -2.6mm (95% CI: -0.9 to -4.3) and remained unchanged in the OMT group -0.2mm (95% CI: -1.6 to 1.9), (p-value for mean between group difference=0.045) (Table S2).

Change in blood pressure
Office blood pressure as well as the 24h ABPM remained unchanged in both groups at 6 months (Table S3).

Change in DDD
No change was observed in the within-group difference in DDD between baseline and 6 months in both the RDN and OMT group (Table S4). No difference was observed between diuretic DDD and DDD of heart failure drugs between both groups at 6 months (Table S5).

Change in quality of life
Based on the RAND-36 questionnaire, two health summary scores were calculated based on eight health concepts with multi-item scales (Table S6).

**Table S1 Change in laboratory findings**

|  | **RDN** | | **Difference**  **(95% CI)**  **or *p*-value** | **OMT** | | **Difference**  **(95% CI) or *p*-value** | **Mean between-group difference (95% CI)** | ***p*-value** |
| --- | --- | --- | --- | --- | --- | --- | --- | --- |
|  | **Baseline** | **6 months** |  | **Baseline** | **6 months** |  |  |  |
| **Creatinine, µmol/l** | 109 ± 29 | 112 ± 36 | 2.9 (-10.9 to 5.23) | 102 ± 26 | 104 ± 27 | 1.86 (-8.4 to 4.6) | -1.41 (-10.6 to 7.8) | 0.76 |
| **eGFR, ml/min** | 68 ± 17 | 68 ± 20 | 0.05 (-3.2 to 3.1) | 70 ± 19 | 71 ± 21 | 0.14 (-5.2 to 4.9) | 1.96 (-3.81 to 7.72) | 0.50 |
| **Cystatin C, mg/L** | 1.30 ± 0.38 | 1.35 ± 0.47 | 0.05 (-0.13 to 0.03) | 1.16 ± 0.30 | 1.21 ±0.36 | 0.05 (-0.15 to 0.04) | -0.01 (-0.13 to 0.11) | 0.83 |
| **NT-pro-BNP, pg/mL⃰** | 19 [8 - 35] | 20 [9 - 47] | 0.83 | 17 [10 - 24] | 15 [11 - 29] | 0.61 | - | 0.81 |

BNP= brain natriuretic peptide, eGFR*=* estimated glomerular filtration rate, OMT*=* optimal medical therapy, RDN*=* renal sympathetic denervation
*non-normally distributed data

**Table S2 Change in echocardiographic parameters**

|  | **RDN** | | **Difference**  **(95% CI)**  **or *p*-value** | **OMT** | | **Difference**  **(95% CI) or *p*-value** | **Mean between-group difference (95% CI)** | ***p*-value** |
| --- | --- | --- | --- | --- | --- | --- | --- | --- |
|  | **Baseline** | **6 months** |  | **Baseline** | **6 months** |  |  |  |
| **LVEF, %** | 32 ± 7 | 33 ± 7 | 0.7 (-2.7 to 1.3) | 33 ± 9 | 34 ± 10 | 0.6 (-2.5 to 1.3) | 0.07 (-2.8 to 2.6) | 0.96 |
| **LVEDD, mm** | 72 ± 7 | 69 ± 7 | -2.6 (-0.9 to -4.3) | 69 ± 13 | 69 ± 13 | -0.2 (-1.6 to 1.9) | 2.4 (0.1 to 4.8) | 0.045 |
| **LVESD, mm** | 63 ± 8 | 62 ± 8 | -1.2 (-1.2 to 3.5) | 61 ± 15 | 60 ± 15 | -0.8 (-0.9 to 2.3) | 0.4 (-2.4 to 3.2) | 0.77 |
|  |  |  |  |  |  |  |  |  |
| **LA size, mm** | 46 [42 – 50] | 44 [42 – 50] | 0.56 | 45 [43 – 52] | 47 [41 – 53] | 0.63 | - | 0.98 |
| **LA volume, ml** | 41 [33 – 50] | 47 [36 – 65] | 0.59 | 53 [46 – 78] | 54 [44 -82] | 0.31 | - | 0.49 |
| **E, cm/s** | 52 [40 – 80] | 47 [32 – 70] | 0.89 | 62 [45 – 83] | 62 [49 – 71] | 0.67 | - | 0.98 |
| **A, cm/s** | 80 [56 – 100] | 65 [55 -87] | 0.009 | 61 [46 – 79] | 60 [50 – 72] | 0.69 | - | 0.048 |
| **E/A ratio** | 0.80 [0.47 -1.10] | 0.63 [0.45 – 1.29] | 0.41 | 0.80 [0.66 – 0.81] | 0.83 [0.69 – 1.26] | 0.78 | - | 0.60 |
| **DET, ms** | 184 [152 – 213] | 221 [161 – 277] | 0.16 | 197 [151 – 238] | 190 [162 – 225] | 0.28 | - | 0.042 |
| **Em septal, cm/s** | 4.4 [3.6 – 6.6] | 3.7 [3.2 – 5.5] | 0.51 | 4.9 [3.4 – 5.5] | 4.2 [3.7 – 6.0] | 0.85 | - | 0.23 |
| **E/Em ratio** | 12 [9 – 16] | 13 [9 – 16] | 0.80 | 13 [10 – 18] | 11 [10 - 15] | 0.22 | - | 0.37 |

A-wave velocity= peak late diastolic filling velocity, DET= Deceleration time, E-wave velocity= peak early filling velocity, LA= left atrium, LVEF= left ventricular ejection fraction, LVEDD=left ventricular end-diastolic dimension, LVESD=left ventricular end-systolic dimension, OMT= optimal medical therapy, RDN= renal sympathetic denervation.

**Note** A significant change was seen in the A-wave velocity in the RDN-group, with no change in the OMT-group, from 80 cm/s [56 -100] at baseline to 65 cm/s [55 - 87] at 6 months, p=0.009 vs. 61 cm/s [46 -79] to 60 cm/s [50 -72], p=0.67 in the OMT group, p-value for difference between group=0.048. No significant change was seen in other indices for diastolic function.

**Table S3 Change in blood pressure, heart rate and mean arterial pressure**

|  | **RDN** | | **Difference**  **(95% CI)** | **OMT** | | **Difference**  **(95% CI)** | **Mean between-group difference (95% CI)** | ***p*-value** |
| --- | --- | --- | --- | --- | --- | --- | --- | --- |
|  | **Baseline** | **6 months** |  | **Baseline** | **6 months** |  |  |  |
| **Sys ABPM** | 111 ± 9 | 109 ± 10 | -1.8 (-1.4 to 5.1) | 108 ± 9 | 110 ± 10 | 1.7 (-5.0 to 1.6) | 3.56 (-8.04 to 0.93) | 0.12 |
| **Dia ABPM** | 69 ± 6 | 68 ± 8 | -0.8 (-1.9 to 3.4) | 66 ± 5 | 67 ± 4 | 0.6 (-3.3 to 2.0) | 1.40 (-5.04 to 2.23) | 0.44 |
| **Office SBP** | 121 ± 11 | 125 ± 16 | 4.2 (-9.4 to 0.9) | 124 ± 19 | 121 ± 16 | -2.8 (-5.4 to 11.0) | 7.04 (-2.29 to 16.5) | 0.14 |
| **Office DBP** | 75 ± 8 | 74 ± 8 | -0.7 (-3.1 to 4.4) | 75 ± 14 | 71 ± 8 | -3.4 (-0.8 to 7.7) | 2.77 (-2.71 to 8.25) | 0.31 |
| **HR*** | 70 ± 9 | 68 ± 8 | -1.3 (-1.3 to 3.9) | 67 ± 9 | 68 ± 9 | 1.2 (-4.3 to 2.0) | 2.46 (-6.39 to 1.48) | 0.21 |
| **MAP*** | 83 ± 5 | 81 ± 8 | -1.4 (-1.0 to 3.9) | 80 ± 5 | 82 ± 5 | 1.4 (-3.4 to 0.7) | 2.80 (-5.93 to 0.24) | 0.78 |
| **PP*** | 42 ± 8 | 42 ± 9 | 0.5 (-2.2 to 1.3) | 42 ± 8 | 44 ± 9 | 1.5 (-3.6 to 0.5) | 1.05 (-3.63 to 1.53) | 0.42 |

ABPM= ambulatory blood pressure measurement, Dia=diastolic, *HR= heart rate (measured 24hours), MAP= mean arterial pressure,. OMT=optimal medical therapy, PP=pulse pressure, RDN= renal sympathetic denervation, Sys= systolic.

**Note** Mean office systolic blood pressure difference in the RDN group was 4.2mmHg (95% CI: -9.4 to 0.9) at 6 months vs. -2.8mmHg (95% CI: -5.4 to 11.0) in the OMT group, p-value for mean between group difference p=0.14. Additionally, mean systolic 24h ABPM in the RDN group changed at 6 months with -1.8mmHg (95% CI: -1.4 to 5.1) vs. 1.7mmHg (95% CI: -5.0 to 1.6) in OMT group, p-value for mean between group difference p=0.12

**Table S4. Defined Daily Dose (DDD) at 6 months within the groups**

The Diuretic Defined Daily Dose is expressed as the sum of the individual DDD of loop diuretics, thiazide diuretics and potassium-sparing diuretics. The Defined Daily Dose of heart failure drugs is expressed as the sum of the individual DDD of ACE-inhibitors, Angiotensin II-antagonists, β-blockers and diuretics including aldosterone receptor blockers.

|  | **RDN** | | ***p*-value**  **baseline vs. 6M RDN** | **OMT** | | ***p*-value**  **baseline vs. 6M OMT** |
| --- | --- | --- | --- | --- | --- | --- |
|  | **Baseline** | **6 months** |  | **Baseline** | **6 months** |  |
| Total antihypertensive drugs DDD | 4.5 [3.6-6.1] | 4.5 [3.4-6.8] | 0.55 | 4.5 [3.1-6.0] | 4.4 [3.0-5.4] | 0.86 |
| Diuretic DDD | 2.2 [1.3-3.7] | 2.3 [1.0-4.3] | 0.31 | 2.1 [1.0-2.5] | 2.0 [1.0-2.5] | 0.50 |
| Loop Diuretic DDD | 2.0 [1.0-3.5] | 2.0 [1.0-8.0] | 0.59 | 2.0 [1.0-3.5] | 2.0 [1.0-2.0] | 0.19 |
| Heart Failure drugs DDD | 4.5 [3.5-6.1] | 4.1 [3.4-6.8] | 0.71 | 4.2 [3.1-5.4] | 3.9 [3.0-5.4] | 0.86 |

Data was presented as median [interquartile range, IQR]. OMT=optimal medical therapy, RDN= renal sympathetic denervation

**Table S5. Defined Daily Dose (DDD) at 6 months between the groups**

|  | **RDN (*n*=24)** | **OMT (*n*=25)** | ***p*-value** |
| --- | --- | --- | --- |
| Total antihypertensive drugs DDD | 4.5 [3.4-6.8] | 4.4 [3.0-5.4] | 0.25 |
| Diuretic DDD | 2.3 [1.0-4.3] | 2.0 [1.0-2.5] | 0.78 |
| Loop Diuretic DDD | 2.0 [1.0-8.0] | 2.0 [1.0-2.0] | 0.72 |
| Heart Failure drugs DDD | 4.1 [3.4-6.8] | 3.9 [3.0-5.4] | 0.25 |

Data was presented as median [interquartile range, IQR].  **Note** Extensive analyses on DDD at baseline and follow-up demonstrated no significant change in both HF drugs and diuretics at baseline and follow-up in both arms.

**Table S6 RAND-36 questionnaire**

|  | **RDN** | | **p-value*** | **OMT** | | ***p*-value**** | ***p*-value**  **between groups** |
| --- | --- | --- | --- | --- | --- | --- | --- |
|  | **Baseline** | **6 months** |  | **Baseline** | **6 months** |  |  |
| **Physical functioning** | 38 [26 – 69] | 60 [41 – 74] | 0.04 | 65 [46 – 79] | 75 [33 -85] | 0.93 | 0.04 |
| **Social functioning** | 56 [38 – 75] | 63 [62 – 88] | 0.14 | 88 [53 – 100] | 88 [63 – 100] | 0.13 | 0.73 |
| **Role of limitations (physical problems)** | 50 [38 – 85] | 45 [42 – 90] | 0.35 | 75 [6 – 100] | 81 [25 – 96] | 0.63 | 0.17 |
| **Role of limitations (emotional problems)** | 83 [8 – 99] | 68 [61 -80] | 0.88 | 67 [33 – 100] | 77 [24 – 87] | 0.73 | 0.90 |
| **Mental health/vitality** | 70 [58 – 79] | 68 [61 – 80] | 0.64 | 76 [65 – 88] | 84 [52 – 92] | 0.59 | 0.88 |
| **Pain** | 78 [45 – 100] | 88 [67 – 100] | 0.20 | 85 [49 – 100] | 87 [57 – 100] | 0.33 | 0.95 |
| **General health perception** | 33 [25 -50] | 40 [21 – 49] | 0.97 | 40 [30 – 60] | 35 [30 – 60] | 0.67 | 0.42 |
| **Health change** | 38 [6 – 75] | 50 [25- 75] | 0.85 | 50 [45 – 55] | 52 [45 – 75] | 0.74 | 0.98 |

*between baseline and 6 months RDN, ** between baseline and 6 months OMT

Besides a significantly greater change in the physical functioning score in the RDN group as compared to the OMT group (p for mean group difference = 0.04) no differences were found between both cohorts. Also, no change was found in the KCCQ (in which the overall and clinical summary score could be calculated) between the groups.
